# Non-invasive tunnelled catheter reposition (NTCR): A simple and safe method to restore central tunnelled catheter function for haemodialysis

**DOI:** 10.1038/s41598-020-64985-3

**Published:** 2020-05-18

**Authors:** Tomasz Porazko, Jacek Hobot, Marian Klinger

**Affiliations:** 1 Department of Internal Medicine and Nephrology, Institute of Medical Sciences, University of Opole, Opole, Poland; 2 Department of Internal Medicine and Nephrology, University Hospital,, Opole, Poland; 3 Department of General and Vascular Surgery, Institute of Medical Sciences, University of Opole,, Opole, Poland; 4 Department of General and Vascular Surgery, University Hospital, Opole, Poland

**Keywords:** End-stage renal disease, Haemodialysis

## Abstract

Despite all efforts, still many end-stage kidney disease (ESKD) patients are dialysed using a central tunnelled catheter (CTC) as vascular access. When the CTC blood flow becomes ineffective, a number of protocols are advised. However, all of them are time- and cost-consuming. The manoeuvre of a non-invasive tunnelled catheter reposition (NTCR) was introduced to restore the CTC function. NTCR was based on gentle movements of the CTC, with or without a simultaneous flushing of the CTC lines, which resulted in a quick reposition of the CTC tip. This study comprises the analysis of a total of 297 NTCRs, which were performed in 114 patients, thus enabling an effective blood flow after 133 procedures (44.7%).Partially effective blood flow followed 123 procedures (41.4%), and it failed altogether in 41 cases (13.9%). Overall, 86% of conducted NTCRs improved the CTC patency to perform a haemodialysis session. The procedure could be successfully repeated, with a similar result after the first and the second attempt. Complications were observed only after 3.4% of all interventions. The novel NTCR manoeuvre was safe and effective in the majority of the CTC dysfunction episodes. It seemed to reduce fibrinolytic usage, allowed an immediate haemodialysis session commencement, therefore, it might save both the costs and the nursing staff time.

## Introduction

According to registries data, CTCs were used in 68% of the incident and 32% of the prevalent haemodialysis patients in Europe^1^. The 2018 Annual Data Report of the United States Renal Data System (USRDS) showed that over 80% of the US patients started haemodialysis with a CTC, and in 69% of them the catheter was still in use after 90 days^2^. After comparing dialysis patients from 20 countries, catheter usage ranged from 1% in Japan to 45% in Canada^3^. CTC dysfunction was a leading non-infectious complication of this type of vascular access for haemodialysis^4,5^. The recent Canadian Observational Study reported one year and two year episodes, in which CTC dysfunction occurred in 15% and 18% of patients, respectively^6^. It was generally defined as a failure to aspirate the locking solution from the CTC lines, blood flow rate (QB) through the lines of less than 300 ml/min., arterial pressure of less than 250 mmHg, high venous pressure greater than 250 mmHg, as well as the necessity for the CTC lines reversal^7–9^. When this complication occurs repeatedly, it may lead to an ineffectiveness of renal replacement therapy (RRT) with low urea clearance (Kt/V < 1.2 or urea reduction ratio <65%)^7–9^. A number of conservative measures and medical management protocols were established to solve the problem. Firstly, it is advised to flush the CTC lines with normal saline solution, place the patient in the Trendelenburg position, on a patient’s sides or adjustment of a head position, and finally one can connect the CTC lines in a reversed way^7,8^. However, repeated attempts, sometimes performed in an inappropriate way, increase the risk of complications, i.e. the CTC damage or a catheter-related infection (CRI). When all mentioned manoeuvres fail, fibrinolysis protocols (using e.g. TPA or urokinase) are introduced^10,11^. Unfortunately, they are time consuming and often shorten the haemodialysis session, as treatment time frames are commonly narrow in dialysis centres. Moreover, the method is expensive. When pharmacologic aid proves ineffective, it is an indication for interventional methods such as endoluminal brush, fibrin sheath stripping or balloon angioplasty disruption, and, finally, a CTC exchange^12–14^.

The optimal CTC tip location is at thejunction of thesuperior vena cava (SVC) and theright atrium (RA). Sometimes, however, even an anatomical correct a tip position does not ensure the optimal blood flow through the line. During the CTC insertion procedure, under fluoroscopy, the operator is moving directly the CTC tip into and out of the junction, and thus he or she may improve the CTC function^15,16^. When the CTC dysfunction occurs, it is commonly practised to put the patient in the Trendelenburg position or turn their head toward or outward the CTC side^7,8^. These manoeuvres shift the chest and the neck skin, together with the underlying tissue, and create impact on the CTC. The consequence is a temporary dislocation of the tip and its release from its adhesion to the vein wall, the surrounding clot or a fibrin sheath. The same mechanism dislocates the CTC tip in obese patients and women with large breasts when they sit up from supine position^17^.

The ability of the CTC to be moved in the tunnel was utilized for the enhancement of the original manoeuvre used to restore catheter patency; that expansion is called non-invasive catheter reposition (NTCR). In the current paper we present a long-term experience with the use of NTCR in the cohort of HD patients.

## Materials and methods

The study was a retrospective analysis of available records, from 2006 to 2018, of the patients who were treated with haemodialysis in the dialysis units of the Department of Nephrology and Transplantation Medicine, Wroclaw Medical University and Wroclaw University Hospital, and the Department of Internal Medicine and Nephrology, Opole University and Opole University Hospital, in whom CTC failure was diagnosed and the manoeuvre of NTCR was used to restore vascular access function.

### Noninvasive tunnelled catheter tip reposition (NTCR) – description of the procedure

**Figure 1:**
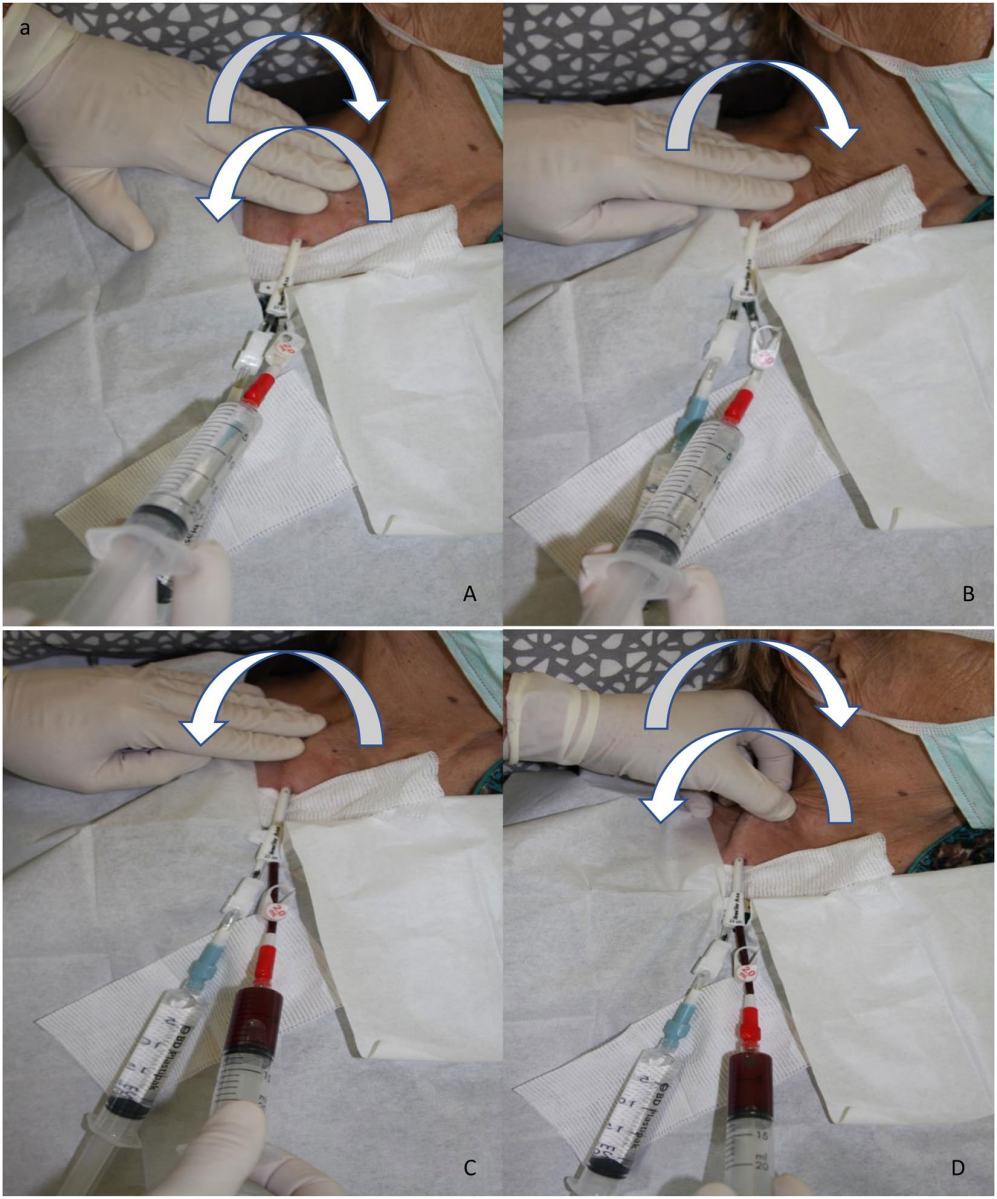
(**A**) Gentle movement of CTC with syringes or lines connected, with skin towards and outwards venotomy, usually 3–4 cm. On the movement in and out patient is asked to cough few times (CTC dressing partially uncovered for presentation). (**B**) CTC is pushed toward vein entering point (left hand, direction showed with an arrow) with simultaneous line flushing (right hand). (**C**) Pressure on CTC is released, CTC line blood flow is achieved and next flushed and aspirated for a few times. (**D**) Alternative option of NTCR, gentle movement in and out of the proximal to the cuff CTC part through venotomy, with simultaneous flushing and aspirating of the line.

The idea behind NTCR was to take advantage of the mobility of the CTC, possible when the skin with subcutaneous tissues is moved, and in consequence, the intravascular end of the CTC may be released from the adjacent tissues. When necessary, it could be supported with CTC lines flushing using physiologic solution. The details of the manoeuvre were as follows. During the preparation for a dialysis session, the dressing of the CTC was removed, and the exit site, together with the surrounding skin area, was cleaned, disinfected and covered with a sterile drape. Both CTC lines were opened, their connectors were disinfected, and two 10 ml syringes were attached to aspirate the locking solution. If blood from the CTC lines failed to be withdrawn freely, maximal suction was produced with the attached syringe and the line was clamped. Routine flushing with 0.9% saline solution was applied, and when this was ineffective, CTC dysfunction was diagnosed. Thereafter, the patient was informed in detail about all the issues regarding the NTCR manoeuvre, especially about the potential complications and discomfort which the manoeuvre may produce for a short period of time (usually for a few seconds – the feeling of pressure or even pain in the chest and neck area, appearing with every attempt at performing NTCR). Every patient was given an alternative option according to local protocol, i.e. locking the CTC lines with fibrinolytic agent at first. After obtaining consent, NTCR was carried out. All steps were performed with strict adherence to standard local sterile requirements. The necessary equipment consisted of two 20 ml Luer lock syringes, a 250 ml bag of 0.9% saline solution, disinfectant wipes, and a sterile cover. The proper equipment to record the patient’s heart rate, blood pressure, body temperature and blood oxygen saturation were available at their bedside. First, one hand was placed along the subcutaneous tunnel and the CTC body was gently moved, under the skin towards a neck, to the vein entering point, usually along the distance of 3–4 cm. As the CTC was moved in and out, the patient was asked to cough a few times (Fig. 1A). When the first step was ineffective, the gentle move of the CTC, as described above, was repeated, accompanied by the flushing of both CTC lines with 0.9% saline solution at a maximum point in and out (Fig. 1B,C). Alternatively, the part of the CTC proximal to its cuff could be gently pulled out and pushed in, with a fold of skin, together with normal saline flushing (Fig. 1D). NTCR was repeated for a few times until the moment when blood could be aspirated and infused freely through the CTC line. The procedure was usually repeated up to 3 times and the overall duration of the manoeuvre was less than 10 minutes. After every attempt the patient was asked for permission to continue the proceedings. There was formally no pain assessment (i.e. visual analogue scale) done after the procedure. Every patient was asked if he would agree, should NTCR be necessary again and his answer was recorded in the patient’s chart. After the failure of NTCR was confirmed, local protocol was implemented, i.e. fibrinolysis protocol, high concentrated heparin instillation, endoluminal brushing, CTC tip stripping or finally CTC guidewire exchange.

The indication for NTCR was CTC dysfunction, defined as the following circumstances: its inability to aspirate the locking solution from both lines, when the blood flow was (QB) < 200 ml/min with arterial pressure <−250 mmHg, as well as when venous pressure >250 mmHg did not improve with conservative measures (i.e. the changing of the patient’s body or head position, normal saline flushing).

Contraindications for the method were: the patient’s refusal, any type of an active or a suspected catheter-related infection (CRI), bleeding or leakage from the tunnel observed during the CTC lines flushing, as well as any damage to the lines, cuffs or connectors. In addition, NTCR could be performed exclusively in the CTCs inserted using the percutaneous method, as in principle, an open surgical method requires placing vascular sutures at the venotomy site, which makes it impossible to perform the manoeuvre.

An early CTC dysfunction was recognized when it occurred within the first week after the CTC insertion; the other cases were treated as a late dysfunction. The aim of NTCR was to restore CTC function, which was defined as the effective blood flow (EQB) through the arterial line of ≥300 ml/min, with arterial pressure ≥250 mmHg and venous pressure ≤250 mmHg. When the achieved blood flow through the arterial line was <300 ml/min but ≥200 ml/min, or when dialysis was performed with reversed lines through the venous line with pre- and post-pump pressures as described before, this procedure was considered to be partially effective (PEQB). All interventions completed without EQB, and even PEQB, were considered to be NTCR failure. When PEQB was achieved, the CTC function was evaluated again after the completion of the dialysis session. When the CTC appeared to be dysfunctional, the same interventions as in the case of the NTCR failure were applied.

The patients’ records were analysed with regard to complications which occurred during the NTCR procedure (i.e. a patient’s condition and complaints) and within following two weeks. Any type of CRI diagnosed during the observation time frame after the last manoeuvre attempt were defined as NTCR-related. The cases that required other, especially mechanical, interventions and the CTC replacement, were excluded. Data were also analysed with regard to other complications that were observed immediately or within two weeks after the last NTCR, which may be related to the manoeuvre, i.e. any damage to the subcutaneous tunnel of the CTC and the CTC cuff dislocation, as well as the hub or the line damage.

A total of 373 NTCRs were performed in 187 patients altogether; however, complete records were available only for 297 procedures in 114 patients. The median age of the individuals under study was 66.5 years (57.0; 72.0 years), and there were 52 (45.6%) males and 62 (54.4%) females among the patients. The observation time for all patient groups was a median of 22.5 months (9.0; 64.75 months). The demographic characteristics of the cohort was presented in Table 1. Sixty (52.6%) patients were dialysed via the CTC as a bridge vascular access before an arteriovenous fistula (AVF) or graft (AVG) maturation; 13 (11.4%) patients were scheduled for peritoneal dialysis catheter implantation or living donor kidney transplantation. In 41 (36.0%) patients, CTC was the only possible option for vascular access. All the CTCs were inserted under US guidance with previous anatomical marking. In the case of a previously complicated CVC or CTC insertion, fluoroscopy was also used. Fluoroscopy guidance was routinely used for the insertion of both Medcomp ® Tesio® and a Ash-Split™ CTCs. However, proper flow through the CTC was superior to the tip location, evaluated in semi-sitting position as would be similar and comfortable for a patient during a haemodialysis session. Most of CTCs were of the double lumen, single line type, and the right jugular vein was the main CTC placement position. Seventy-two (62%) patients had their CTCs locked with high concentration heparin (5000 U/ml), 21 (18%) patients with low concentration heparin (1000 U/ml), 11 (10%) patients with taurolidine, 4 (4%) patients with citrates and 6 (6%) patients had their CTCs secured with Tego® caps system. The type of lock solution had no impact on NCTR effectiveness.

**Table 1:** Characteristics of the group under study.

Characteristic		Pts n	Total group
Age (median, years)		114	66.50 (57.00; 72.00)
Sex	Female	62	54.4%
	Male	52	45.6%
CKD cause	Diabetes mellitus	33	28.9%
	Hypertension	19	16.7%
	Unknown	19	16.7%
	Cancer	18	15.7%
	Glomerulonephritis	15	13.2%
	Other	7	6.0%
	Adult polycystic kidney disease	3	2.6%
VAS/RRT options	Awaiting AVF/AVG	60	52.6%
	Awaiting KTx or PD	13	11.4%
	Not suitable for AVF/AVG/KTx	41	36.0%
CTC type	Dual lumen	84	73.7%
	Tesio	30	26.3%
CTC location	Left femoral	3	2.6%
	Left jugular	31	27.2%
	Left subclavian	2	1.8%
	Right femoral	3	2.6%
	Right jugular	73	64.0%
	Right subclavian	2	1.8%

AVF – arteriovenous fistula, AVG – arteriovenous graft, CKD – chronic kidney disease stage 5, RRT – renal replacement therapy, PD – peritoneal dialysis, KTx – kidney transplantation, and VAS – vascular access status.

Out of 114 patients, 10 patients were treated with low molecular weight heparin, 8 patients with vitamin K antagonists, and 52 patients with aspirin due to cardiovascular indications.

### Ethical approval

All procedures under study were aimed at restoring vascular access function for haemodialysis and life support therapy. These procedures were conducted in accordance with the ethical standards of the Declaration of Helsinki from 1964 and its later amendments. The study was approved by the Bioethics Commission of Opole Public Medical Higher Professional School. Informed consent was obtained from all the individual patients before every procedure. The patients presented in the images providing illustration for NTCR have given their written consent for publishing the materials.

### Statistical analysis

The data are presented as absolute frequencies (*n*) and percent values of the total group for nominal variables. A continuous variable (age) was tested for normality with the Shapiro-Wilk test and, due to the lack of normal distribution, it was presented as the median and interquartile range (*IQR*: *Q1*; *Q3*). The remaining variables had an original scale and are presented as the medians (*Q1*; *Q3*). The comparisons of number of sessions between the different groups (number of procedures, sex, disease type and position) were conducted with the U-Mann-Whitney test or Wilcoxon paired test, as appropriate. Additionally, the median difference (*MD*) between the subgroups analysed with a 95% confidence interval was calculated. The relation between the number of sessions and age for each procedure was evaluated with the Spearman correlation coefficient. For the number of procedures by sessions, a Kaplan-Meier survival curve was generated. Additional curves stratified by age, position and chronic kidney disease were produced, including the calculation of log-rank test, to assess the impact of these variables on survival. To generate curves stratified by age, 3 age groups were created: 35–50 years, 51–60 years, and ≥61 years. Analyses were carried out with the use of statistical software R (version 3.5.2), the R Foundation for Statistical Computing, c/o Institute for Statistics and Mathematics, Wien, Austria.

## Results

**Figure 2:**
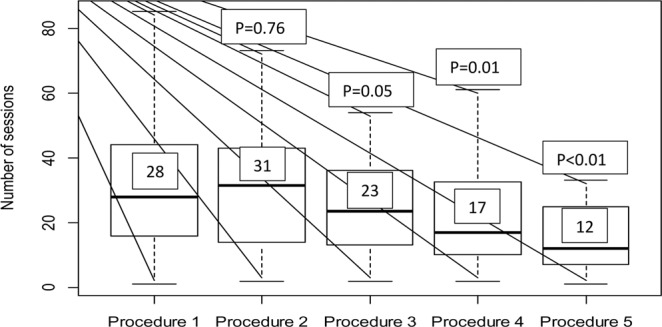
Boxplot of median hemodialysis sessions number of HD comparison, after first and subsequent NTCR procedure.

As mentioned before, a total of 373 NTCRs were performed in a total of 187 patients; available, and the complete records of 297 NCTRs performed in 114 patients were subject to analysis. The contraindications for NTCR were found in 27 patients and hence the procedure was not performed. The particular causes were as follows: catheter-related infection – 22 patients, connector damage – 3 patients, and line damage – 2 patients. There was no patient who refused to have NTCR performed for the first time or who objected to a subsequent attempt when it was necessary. The demographic and clinical characteristics of the study group was presented in Table 1. It is worth emphasizing that most patients were older than 65 years (median age 66.5 years). Regarding ESKD aetiology, 28.9% cases were attributed to diabetes, 16.7% to hypertension, and 15.7% to morbidity related to cancer and its complicated therapy. For 41 (36%) patients, CTC was the only possible vascular access option. The two main types of CTC used were a double-lumen CTC (84 cases; 73.7%) and a Tesio CTC (30 cases; 26.3%). The frequency of a non-infection-related CTC dysfunction, in the entire cohort, was of 1.5 episodes per 1000 catheter days. The results of NTCR were presented in Table 2. A total of 297 NTCRs (2.5 procedures per patient (p/p) were performed in 114 patients. The median number of dialysis sessions performed from the time of CTC implantation to the moment of CTC dysfunction was 68 (2-197). In 21 (18.4%) cases, intervention was undertaken due to an early CTC dysfunction. The remaining 276 (81.6%) episodes were related to a late CTC dysfunction. Only one attempt at NTCR was required in 32 (28%) patients, 2 interventions were carried out in 30 (26%) patients, and 3 or more procedures were necessary in 52 (46%) individuals. Effective blood flow (EQB) was achieved after 133 (44.7%) interventions, PEQB was achieved after 123 (41.4%) NTCRs, and failure occurred in 41 (13.9%) attempts (Table 2.). The success rate of NTCR, measured with EQB and PEQB, dropped at the third and later procedures (Table 3). The first manoeuvre brought back EQB in 52.6% of cases and PEQB in 46%. Compared with that, after the third attempt EQB and PEQB were achieved equally in 25% of cases (Table 3). After the first NTCR attempt, in 114 patients the median number of properly conducted dialysis sessions was 28, after the second procedure the positive outcome included 31 sessions in 82 patients, and after the third attempt – it reached the number of 23 dialyses in 52 patients (Table 2.). The subsequent manoeuvres were significantly less effective with a decrease in the number of haemodialysis sessions after the fourth and the fifth procedure in comparison with the first one (MD = −11.0 *CI*_*95*_ [−17.0; −2.0], *p* = 0.012 and *MD* = −16.0 *CI*_*95*_ [−24.0; −5.0], *p* = 0.003, respectively; Table 4, Fig. 2). However, a similar comparison conducted only for patients who underwent all five attempts of the procedure (*n* = 15) did not show a statistically significant change in the number of sessions over time (Table 4).

**Table 2:** NTCR results in total group of patients.

Characteristic	Value	n	Total group
CTC dysfunction episodes		297	
Early dysfunction		21	18.4%
Late dysfunction		276	81.6%
Total number of procedures		297	100%
EQB		133	44.7%
PEQB		124	41.7%
Failure		41	13.8%
**Number of effectively performed haemodialysis sessions after procedure**		**n**	**(median number of HD sessions)**
	Procedure 1	114	28.00 (16.25; 43.75)
	Procedure 2	82	31.50 (14.75; 43.00)
	Procedure 3	52	23.50 (13.00; 36.00)
	Procedure 4	34	17.00 (10.00; 32.50)
	Procedure 5	15	12.00 (7.00; 25.00)

Data presented as % of total group or median (Q1, Q3). CTC – central tunnelled catheter, EQB – effective blood flow, PEQB – partially effective blood flow.

**Table 3:** Results of NTCR, after subsequent attempts, in all patients.

NTCR att.	NTCR (n)	EQB (n)	%	NEQB (n)	%	F	%	CTC ex (n)	%
1^st^	114	60	52.6	46	40.3	8	7	4	3.5
2^nd^	82	42	51.2	29	35.3	11	13.4	8	9.7
3^rd^	52	13	25	26	50	13	25	10	19
4^th^	34	11	32.3	18	52.9	5	14.7	4	12
5^th^	15	7	46.6	4	26.6	4	26.6	5	33
Total	297	133	44.7	123	41.4	41	13.8	31	10

NTCR att. – NTCR attempt, EQB – effective blood flow, NEQB – non-effective blood flow, F - failure to restore CTC function, and CTC ex – central tunnelled catheter exchanged.

**Table 4:** . Comparison of hemodialysis sessions number after first and subsequent NTCR procedures.

	All patients included in a given procedure^a^			Only patients who had all 5 procedures^b^		
	*n*	Number of sessions	*P*	*n*	Number of sessions	*P*
Base (Procedure 1)	114	28.0 (16.0; 44.0)	—	15	19.0 (6.0; 26.0)	—
Procedure 2 vs. Procedure 1	82	31.0 (14.8; 43.0)	0.76	15	23.0 (5.0; 35.0)	0.20
Procedure 3 vs. Procedure 1	52	23.0 (13.0; 36.0)	0.05	15	14.0 (8.0; 36.0)	0.42
Procedure 4 vs. Procedure 1	31	17.0 (10.0; 32.0)	0.01	15	15.0 (8.0; 27.0)	0.22
Procedure 5 vs. Procedure 1	15	12.0 (7.0; 25.0)	<0.01	15	12.0 (7.0; 25.0)	0.49

Data presented as median (Q1; Q3); ^a^groups compared with the U-Mann-Whitney test and ^b^groups compared with the Wilcoxon paired test.

An unsuccessful NCTR attempt was an indication for measures carried out in accordance with the local protocol, as mentioned before. In 31 (10.4%) cases the CTC had to be exchanged (Table 3). An indication for that was a failure of all the methods to restore any CTC patency (28 [90%] cases) or prolonged PEQB with insufficient dose of dialysis (3 [10%] cases; Table 3). In a multivariate model, the only factor significantly lowering the success rate was the number of the attempts, i.e. the third and further NTCR procedure. There was no relation between the success or failure of NTCR and the age, gender, the type of a CTC comorbity, CTC location, the use of anticoagulation or antiplatelet therapy, as well as the type of the CTC locking solution.

The NCTR manoeuvre appeared to be safe. The patients communicated mainly the nonspecific symptoms of a sensation of warmth or being short of breath and feeling dizzy – 142 cases (Table 5). None of these lasted longer than for a few seconds, and it did not result from the readings of abnormal vital signs as heart rate, blood pressure, respiratory rate, saturation, body temperature, glucose level or deviation in neurological condition. Complications were noticed after 13 interventions (3.4%). Five cases of supraventricular tachycardia (SVT) of focal atrial type (AT) occurred, without hemodynamic instability; three of them were resolved with the Valsalva manoeuvre and 2 patients required a single dose of beta blocker. There were two cases of tunnel hematoma which absorbed without additional procedures. Two cases of catheter-related infection were observed. One case with systemic symptoms, i.e. fever, feeling unwell; however, no abnormal blood test results were present. Second patient was diagnosed a CTC exit site infection without systemic symptoms. Both cases were resolved with the use of an antibiotic treatment. Two (0.6%) CTCs had to be exchanged due to cuff dislocation, and in the other two (0.6%), the CTC broken connectors were repaired with sets of proper ones. All complications were observed during the first two weeks after the procedure.

**Table 5:** NTCR-related side effects and complications.

Characteristic	Value	n	In all NTCRs
**Complications (episodes)**			
	Nonspecific symptoms	142	48.0%
	Supraventricular tachycardia	5	1%
	Tunnel haematoma	2	0.6%
	Catheter-related infection	2	0.6%
	Connector rupture	2	0.6%
	Cuff dislocation	2	0.6%

As it was mentioned before, there was no formal pain assessment (i.e. visual analogue scale) done after the procedure. However, all the patients expressed exclusively the feeling of a “mild pressure”, “ mild pain “ or even no complaints whatsoever. There was no patient who – after the NTCR attempt – admitted that he/she would not agree to have it done again.

## Discussion

Central tunnelled catheter dysfunction is the leading non-infectious complication causing the patient’s inability to obtain an effective dialysis treatment^18^. The non-invasive tunnelled catheter tip reposition (NTCR) manoeuvre presented in this paper may offer the dialysis staff an additional cost-effective tool to restore CTC function before the administration of fibrinolytic agents. Successful NTCR enables an immediate start of the dialysis, saving time and costs. The presented results include 297 cases of NTCR procedures performed in 114 patients. The cohort reflects the characteristics of the current worldwide dialysis population with a growing number of elderly and frail patients (median age 66.5 years), with diabetes, hypertension and cancer as the leading causes of ESKD. Collectively, 156 (86%) NTCR interventions allowed an immediate start of the dialysis session, with effective blood flow after 133 (44%) procedures and partial restoration after 123 (42%) manoeuvres. This result is quite satisfactory, when compared with the effectiveness of fibrinolytic agents^19^. The first instillation of alteplase restored full catheter patency in 72% patients, whereas after first NTCR the complete (52.6%) and partial catheter patency restoration (40.3%) was achieved in 92.9% cases. Moreover, NTCR could be successfully repeated when necessary. The success rate of the first and the second attempt was similar, however it dropped significantly after the third and later attempts. The patency of NTCR effect was also similar after the first and the second manoeuvres, extending to 28 and 31 haemodialysis sessions, respectively, with the loss of longevity after three or more attempts. It should be emphasized that the NTCR manoeuvre is suitable exclusively for percutaneously inserted CTCs. An open surgical CTC insertion method requires vascular sutures at the venotomy site, which potentially may cause vein wall rupture when NTCR is applied. The NTCR manoeuvre appeared to be safe. The reported complaints included mostly a nonspecific feeling of warmth, shortness of breath, or dizziness, which lasted for a few seconds. The separation of the CTC body movements from the flushing of theCTC lines reduced discomfort to a patient and provided an opportunity to detect possible side effects. It is crucial to limit the number of syringe disconnections to a minimum in order to reduce the risk of catheter-related blood stream infections. Generally minor complications occurred only after 13 (3,4%) interventions. The NTCR manoeuvre might extend a paramount of available tools for CTC patency restoration, which could be of particular value for the patients awaiting live donor transplantation and elderly patients with limited life expectancy^20,21^. The application of fibrinolysis remained exclusively for failed NTCR cases.

## Conclusions

The non-invasive tunnelled catheter tip reposition (NTCR) manoeuvre appeared to be a novel, effective and safe tool for the restoration of central tunnelled catheter (CTC) blood flow in the vast majority of malfunction cases. NTCR may enable an immediate start of haemodialysis session, avoiding treatment delay. Its application might reduce the usage of fibrinolytic agents, and save costs and nursing staff time.
